# Defect detection by multi-axis infrared process monitoring of laser beam directed energy deposition

**DOI:** 10.1038/s41598-024-53931-2

**Published:** 2024-02-16

**Authors:** T. Herzog, M. Brandt, A. Trinchi, A. Sola, C. Hagenlocher, A. Molotnikov

**Affiliations:** 1 School of Engineering, Centre for Additive Manufacturing, RMIT University, Melbourne, VIC 3000 Australia; 2 CSIRO Manufacturing, Clayton, VIC 3168 Australia

**Keywords:** Mechanical engineering, Imaging techniques

## Abstract

Laser beam directed energy deposition (DED-LB) is an attractive additive manufacturing technique to produce versatile and complex 3D structures on demand, apply a cladding, or repair local defects. However, the quality of manufactured parts is difficult to assess by inspection prior to completion, and parts must be extensively inspected post-production to ensure conformance. Consequently, critical defects occurring during the build go undetected. In this work, a new monitoring system combining three infrared cameras along different optical axes capable of monitoring melt pool geometry and vertical displacement throughout deposition is reported. By combining multiple sensor data, an automated algorithm is developed which is capable of identifying the formation of structural features and defects. An intersecting, thin-walled geometry is used to demonstrate the capability of the system to detect process-induced porosity in samples with narrow intersection angles, which is validated using micro-CT observations. The recorded results indicate the root cause of this process-induced porosity at the intersection, and it is shown that advanced toolpath planning can eliminate such defects. The presented methodology demonstrates the value of multi-axis monitoring for identifying both defects and structural features, providing an advancement towards automated detection and alert systems in DED-LB.

## Introduction

Metal laser beam directed energy deposition (DED-LB/M) is an additive manufacturing (AM) process that produces metallic parts through laser melting of feedstock and substrate. Depending on the system being used, a laser is directed onto a metallic substrate forming a liquid melt pool. Feedstock material, in either powder or wire form, is then directed into the laser path and creates a liquid melt pool which rapidly solidifies into a bead. As the laser and feedstock are moved relative to the substrate, a bead of metal is deposited which can be further built upon to create claddings, repair metallic components, or create entire three-dimensional structures.

DED-LB/M, along with other metallic AM processes, is proliferating throughout research and industry, resulting in rapid technological development for both the manufacturing equipment and the applications for which it is being used. As this development continues, there is an increasing demand for tighter control over the AM process to ensure the repeatability and quality of the manufactured products. Industries, such as the medical, energy, and aerospace sectors, now routinely use additively manufactured components at commercial scales, and as such, require parts to be consistent and reliable in their performance^1–3^. However, metal AM is a high-energy process with a high degree of inherent variability. Small fluctuations in feedstock and its delivery, heat accumulation, part geometry or other print conditions can quickly accumulate, resulting in defects or leading to variability in part properties^4,5^.

The occurrence of defects and performance inconsistency are persistent obstacles to the further adoption of AM processes into industry. Parts produced for performance-critical applications require extensive testing and inspection before use, adding significant cost to the value of the part. Specifically, internal defects are often detected and characterised through the use of destructive testing or by non-destructive methods, such as micro X-ray Computed Tomography (micro-CT)^6^. Both methods can be slow and costly, only detecting the presence of defects once the part has been built.

One method to minimise the occurrence of defects and variability is to use well understood processing parameters, and process maps are frequently created to facilitate this. Process maps can be used to identify and avoid regimes that may commonly result in various defect types. For instance, it is well documented that low energy input can lead to lack of fusion defects^7,8^. However, it has been suggested by Chen et al.^9^ that lower traverse speeds can also introduce lack of fusion porosity by creating a coating of sintered particles that do not fully melt in future layers. Similarly, high energy input can produce keyholes in the melt pool, which may lead to the formation of porosity^10^. While beneficial, process maps tend to be time-consuming to produce, reliant on computational solutions, and are often affected strongly by part geometry or machine-specific parameters^11^, nor do they actually guarantee defect-free components^12^.

To address the difficulties in controlling part quality and to identify potential issues at an early stage of the build process, many researchers and industrial operators are turning towards the growing field of in-process or in-situ monitoring^13–16^. This refers to the use of one or more sensors that monitor the additive manufacturing process as it takes place, allowing users to better understand the physics and interactions that occur during metal additive manufacturing processes which may inform how they relate to defect formation^17^. Through this approach, it has recently become possible to detect the formation of defects during the AM process from recorded signals^13,18, 19^.

There are a variety of approaches to vision-based monitoring of DED-LB/M in literature. For instance, Jeon et al.^20^ used a mid-wave (3–5 μm) infra-red (IR) camera, which was coaxially coupled to the optical train, and a line scanner mounted 120 mm behind the deposition nozzle to measure the deposit profile of the Stainless Steel (SS) 316L samples. However, the positioning of the laser scanner limits the complexity and size of samples that can be investigated to be predominantly linear in nature and small enough to allow for necessary over-travelling.

Off-axis vision-based monitoring of DED has also been conducted to observe both temperature and layer height. Hagenlocher et al.^14^ used an IR camera in a stationary position and were able to measure the temperature of an entire side of an AlMg5 rectangular tube, enabling analysis of the heat accumulation within the part. However, this arrangement only permits a single field of view during the entire build process. In studies by Borovkov et al.^21^ and Lu et al.^22^, the height of deposited layers was measured using off-axis cameras but required a projection of a line laser onto the track behind the melt pool, limiting the monitoring directionality.

Height measurements can also be conducted coaxially using Optical Coherence Tomography (OCT)^23,24^ or laser triangulation^25,26^. The coaxial nature of these systems enables measurements of surface height from the melt pool and can be used irrespective of traverse direction. However, these systems require an additional laser probe to be coaxially coupled into the optical train, which is then monitored by a coaxially coupled sensor, requiring appropriate optics to be installed if not present. Moreover, laser wavelength and optics design must be carefully considered to avoid interference from either the process laser or the emissions from the process zone^23^. The effects of feedstock entry (especially in powder form, which introduces significant signal distortion), surface condition, and process plume must be evaluated on a case-by-case basis for these monitoring techniques^25^.

In this work, to maximise the advantages of both off- and on-axis monitoring methods, a multi-axis monitoring system was implemented using one coaxial and two off-axis infrared cameras to maintain as much independence from deposition direction as practical. Using this approach, multiple geometric factors were extracted from the video feed, including commonly monitored signals such as melt pool area, length and width^27,28^. However, of greatest interest for this study, the position of the melt pool centroid was tracked by the off-axis cameras to determine the changes in the surface height and enable layer-height monitoring continuously irrespective of traverse direction.

A set of experiments was designed to monitor the deposition of Ti6Al4V powder into thin-walled geometries with angled intersections. This arrangement captures views of the melt pool from the top, front, and side at a fixed observation angle to provide a holistic picture of the process zone. The implementation of multiple-axis observation is shown to provide robust measurements and feature identification. Tracking the melt pool between the multiple cameras allows geometric factors and vertical height displacement to be monitored during multi-directional deposition without interruption. This system is designed to be adaptable for different IR cameras and existing deposition equipment. The capability of this approach for detection of geometric deviations through the vertical displacement monitoring is demonstrated, identifying the different modes of displacement that are indicative of material depletion at the intersection and excess material at the laser turn-around locations.

## Materials and methods

### Material and processing

All experiments performed in this study were conducted on the TRUMPF TruLaser Cell 7020 (TRUMPF SE + Co. KG, Ditzingen, Germany). This 5-axis system delivers a powder feedstock by coaxial nozzle into the melt pool formed by a 1030 nm laser supplied by a TRUMPF TruDisk 3001 laser module. This solid-state Yb:YAG laser provides a continuous beam with Gaussian distribution and power output of 80–3000 W; the beam is transported by fibre optic cable. The coaxial nozzle with a 12 mm stand-off distance was utilised in all experiments.

The feedstock powder used is ASTM grade 5 Ti6Al4V supplied by ECKART TLS GmbH (Hartenstein, Germany). The particles are produced by gas atomisation in Argon and are classified as spherical with a size distribution of 45–90 μm. Samples were deposited onto sandblasted Ti6Al4V substrates with a minimum thickness of 10 mm. Other important experimental parameters used are listed in Table 1.

**Table 1:** Important material and process parameters used for deposition of intersecting geometry samples.

Parameter	Value/description
Laser spot diameter	1.5 mm
Laser power	480 W
Traverse speed	800 mm/min
Track spacing	0.75 mm
Powder feeder rotation rate	2.5 rpm
Powder feed rate	2.3 g/min
Carrier gas	Helium
Helium flow rate	10 L/min
Shielding gas	Argon
Argon flow rate	16 L/min
Nozzle type	Coaxial
Stand-off distance	12 mm

**Figure 1:**
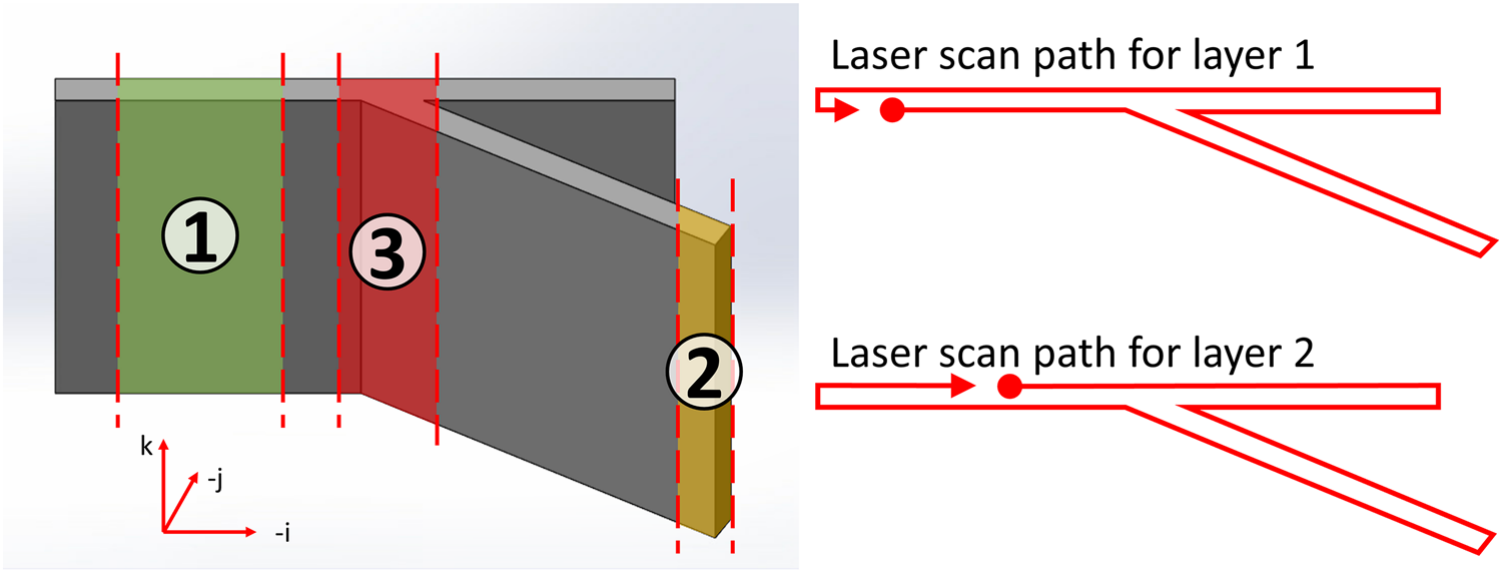
CAD model showing the geometry of Intersecting wall samples where the intersection angle is 30° and the design height is 20 mm. The scan paths for the first two layers (fillets not shown) are depicted to illustrate the deposition strategy. The coordinate axes for the laser deposition system are also shown. The locations of interest are highlighted here with numbers denoting: 1. Wall mid-regions; 2. Wall turn-around points; 3. Wall junction.

A series of nine intersecting thin-wall geometries were produced for this study at three different design heights, 5, 10 and 20 mm, with intersection angles of 90°, 60° and 30° to represent common geometric shapes. An example of this geometry is depicted in Fig. 1. The machine toolpath was generated from a CAD model with the slicing software DCAM by SKM Informatik (Schwerin, Germany), resulting in an alternating scan direction for each layer while the start and endpoints progressed around the perimeter of the build. Due to the acceleration and deceleration of the laser at the start point, the resulting melt flow can cause the formation of a bulge if each layer begins in the same place^29,30^. Hence, the scan strategy chosen mitigates the cumulative effect of a constant start point and traverse direction. A further three samples were later manufactured using a modified toolpath, one at each intersection angle, and will be discussed in more detail in “Camera limitations” section.

It is hypothesised that the design will create three primary regions with different thermal histories producing different geometrical features. These build portions are: (1) the mid-regions of the thin walls where the laser moves in a straight line and deposition can be assumed to be in a steady state mode; (2) the ends of each wall where the laser must slow and turn around before resuming its linear tracing of the design, resulting in an increased residence time at these locations; and (3) the junction region where the walls intersect. The wall junction is of particular interest as the laser passes through this region three times, compared to the two times it passes every other location in the part.

The sample designations are of the form IW1, where the letters stand for ‘Intersecting Wall’, and the number relates to the design height and angle of intersection. They are provided in Table 2.

**Table 2:** Designations of samples based on the design height and intersection angle.

Sample height	90°	60°	30°
20 mm	IW1	IW4	IW7
10 mm	IW2	IW5	IW8
5 mm	IW3	IW6	IW9
20 mm—modified toolpath	IW1-Mod	IW4-Mod	IW7-Mod

### In-process monitoring

**Figure 2:**
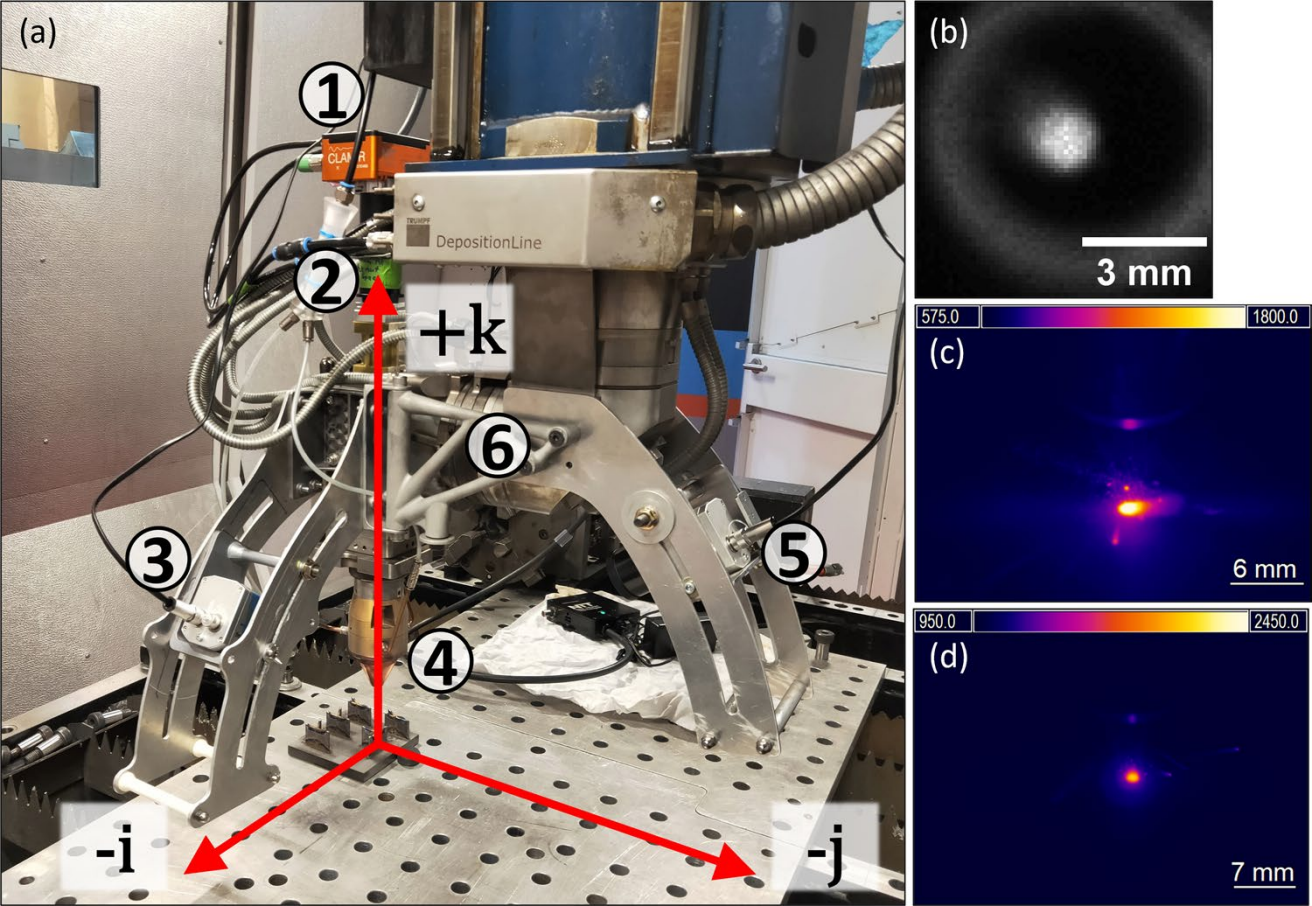
Experimental setup and example images from the manufacture of a 30° intersecting wall sample. (**a**) Installation of thermal cameras on TRUMPF deposition head with machine coordinate axes superimposed. The primary components are numbered: (1) CLAMIR camera with water cooling and data cable connected. (2) Coaxial mounting port for CLAMIR camera. (3) Optris PI 05M thermal camera, with data cable, at 55° to the k-axis. Attached via side-mounted bracket. (4) Coaxial powder nozzle for DED-LB/M. (5) Optris PI 1M thermal camera, with data cable, at 55° to the k-axis. Attached via rear-mounted bracket. (6) Stabilisation brace for side-mounted bracket. (**b**) Greyscale image obtained from CLAMIR camera. (**c**) Thermographic image obtained from Optris PI 1M camera. (**d**) Thermographic image obtained from Optris PI 05M camera.

The build process is monitored using three infrared cameras, which were attached to the deposition head of the TRUMPF and moved with it during deposition. The experimental setup includes Optris PI 05M and Optris PI 1M thermal cameras (Optris GmbH, Berlin, Germany) positioned on a side-mounted bracket and a coaxial infrared imaging camera, Control for Laser Additive Manufacturing with Infrared (CLAMIR), supplied by NIT (New Infrared Technologies, SL, Madrid, Spain)). The arrangement of these cameras is shown in Fig. 2a, with examples of the images obtained by the cameras in Fig. 2b–d. This arrangement allows for one off-axis camera to monitor the longitudinal profile of the melt pool while the other monitors the front-on profile, or vice versa.

#### Geometric corrections

**Figure 3:**
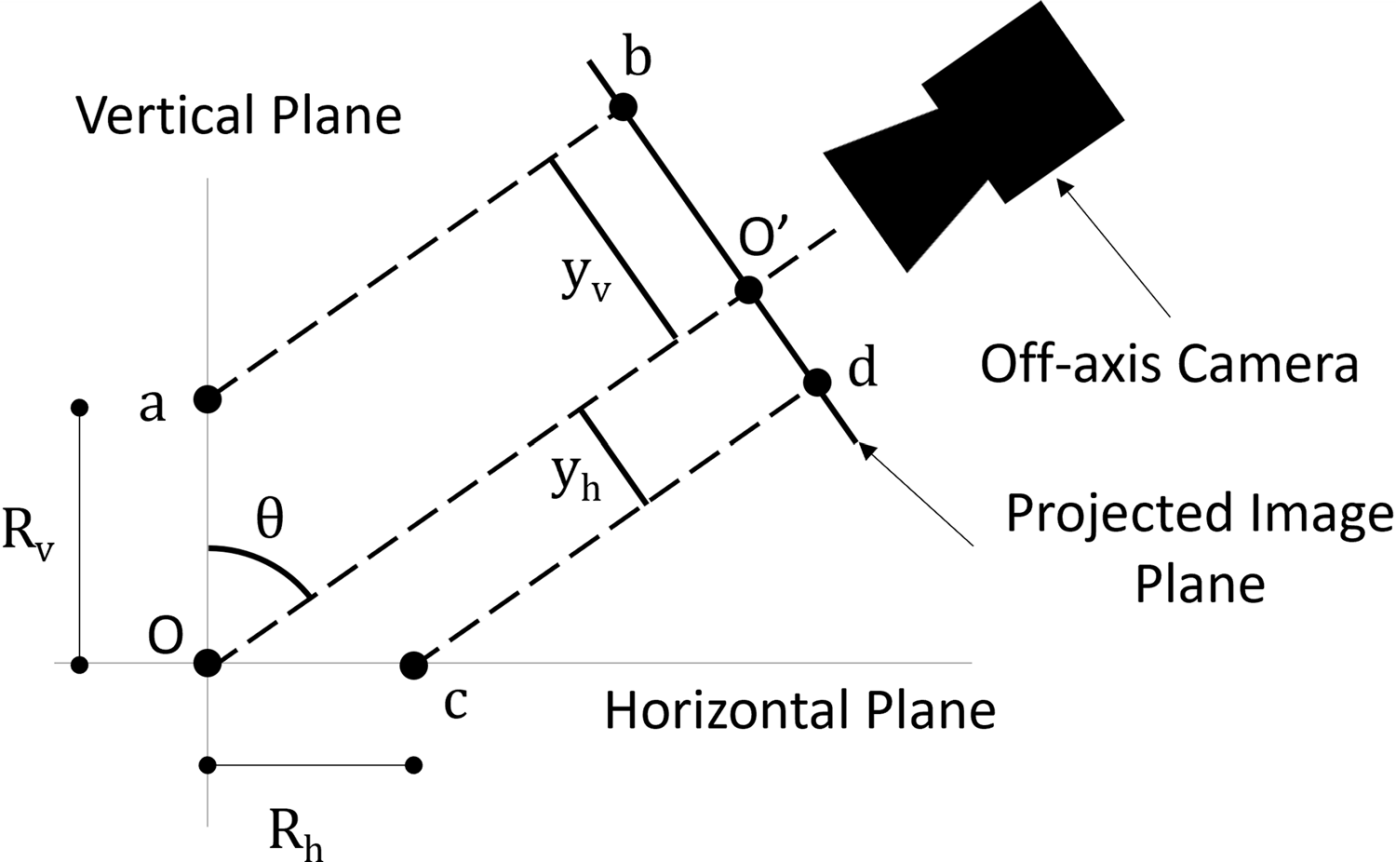
Projection of vertical and horizontal planes onto image plane for off-axis cameras. Real points a and c are projected to image points b and d, respectively, provided that a parallel ray assumption can be made. The focal point of the laser is taken to be the origin, O, which is mapped to point O’ in the image plane. Hence, the real distances in the vertical, Rv, and horizontal, R_h_, planes are mapped to distances in the image plane, y_v_ and y_h,_ respectively. As the laser optical axis does not change, the melt pool can be assumed to be centred about the vertical axis, and changes to the y-axis of the melt pool centroid relate to changes in vertical displacement, while the extent of the melt pool in the image y-axis can be corrected by using the equation for real distance in the horizontal plane.

The perspective of each camera must be considered for monitoring geometric properties. The coaxial camera experiences no perspective distortion and requires no correction factors. However, the two off-axis cameras must undergo a perspective correction to measure vertical displacement and to correct for the pixel aspect ratio. As will be discussed further in “Data processing” section, measurements are obtained through melt pool tracking. Since the deposition optics maintain the same vertical alignment throughout the sample manufacture, the melt pool can be assumed to be centred around the vertical axis. Therefore, changes to the image coordinates of the melt pool centroid in the y-axis of the image can be interpreted as vertical displacement from the initial focal plane. Points in the vertical plane will be projected into the image plane as shown in Fig. 3, and the real distances in the vertical plane, or the k-axis of the deposition system as per Fig. 2a, can be calculated using the trigonometric relation, see Eq. (1),

1 $$\begin{array}{c}{R}_{v}=\frac{{y}_{v}}{\sin\left(\theta \right)},\end{array}$$

where ${R}_{v}$ is the real distance between two points in the vertical plane, ${y}_{v}$ is the distance in the image y-axis and $\theta$ is the angle between the camera’s optical axis and the vertical axis. Similarly, the scaling factor of the pixels in the y-axis of the image can be determined using Eq. (2),

2 $$\begin{array}{c}{R}_{h}=\frac{{y}_{h}}{\cos\left(\theta \right)},\end{array}$$

where ${R}_{h}$ is the real distance between two points in the horizontal plane (the i–j plane of the deposition system), and ${y}_{h}$ is the distance in the y-axis of the image.

#### CLAMIR

Coaxial monitoring of the melt pool is performed using the CLAMIR imaging camera module from NIT. This camera couples into the optical train of the TRUMPF deposition head above the collimating lens, as shown in Fig. 2a, location 1. CLAMIR is a mid-wave infrared camera detecting wavelengths in the spectrum of 1–5 μm on a 64 × 64 pixel PbSe detector with a maximum framerate of 1 kHz. A long-pass filter, ThorLabs (ThorLabs Inc., Newton, U.S.A) FELH1100, is installed to block all wavelengths shorter than 1100 nm and protect the detector from laser reflections. Frames were captured at a frequency of 1 kHz, and a pixel resolution of 9.0 px/mm (111 μm/px) was achieved. While the CLAMIR camera might be used to automatically adjust the laser power to achieve a target width of a melt pool, this function was not used within the present study due to inconsistencies in the obtained results, and instead the generated sensor data was recorded for in-house processing and evaluation.

The camera is equipped with an autoshutter function to prevent thermal drift on the sensor, which would lead to an increasing perceived intensity for all pixels. During the period the shutter is active, approximately 300 ms, the image recorded is blank, but any calculated metrics in the native software are maintained as the last active instance. The autoshutter was set to activate every 30 s during monitoring as internal testing showed minimal thermal drift occurring within this timeframe. Further information can be obtained from the CLAMIR datasheet^31^.

#### Optris PI 1M

The first off-axis camera is the Optris PI 1M camera which monitors wavelengths in the 0.85–1.1 μm range and has a maximum measured temperature range of 450–1800 °C. In this study, the measurement range is 575–1800 °C, which covers the melting range of Ti6Al4V, i.e., 1600–1650 °C^32^. A notch filter, ThorLabs NF1030-45, is installed with this camera to exclude wavelengths of 1030 ± 2 nm, with a full width at half-maximum of 45 nm for the blocking region. This prevents reflected laser wavelengths from damaging the detector or affecting the temperature estimation. In-house designed and manufactured bracket was used to mount the camera and ensure the repeatability of the experiments, see Fig. 2a, location 5. The viewing angle was set to 55° from the vertical axis and resulted in a focal length of 0.3 m. Frames were captured at a frequency of 80 Hz, optical resolution of 382 × 288 px, and a pixel resolution of 11.7 px/mm (85.7 μm/px) was achieved. The Optris 1M camera was aligned with the j-axis of the deposition system and viewed the intersecting geometries in the positive j-direction, perpendicular to the rear of the long wall. Further information can be obtained from the Optris PI 1M datasheet^33^.

#### Optris PI 05M

The second off-axis camera is the Optris PI 05M thermal camera, which is installed on the side of the deposition head using a second bracket, 90° from the PI 1M camera in the horizontal plane, as shown in Fig. 2a, location 3. The inclination angle of this camera was also set to 55° from vertical and had a focal length of 0.2 m. The 05M camera monitors wavelengths in the 500–540 nm range and observed a temperature range of 950–2450 °C. This shorter wavelength band also removes the necessity of an optical filter for protection against the laser light. Frames were captured at a frequency of 80 Hz, optical resolution of 382 × 288 px, and a pixel resolution of 8.3 px/mm (120 μm/px) was achieved. The Optris 05M camera was aligned with the i-axis of the deposition system as per Fig. 2a and viewed the intersecting geometries in the positive i-direction, facing the interior side of the intersection angle. Further information can be obtained from the Optris PI 05M datasheet^34^.

Similarly to the CLAMIR camera, both Optris cameras are equipped with a function to prevent thermal drift, termed the ‘flag’. This is again a physical barrier closed over the optics briefly to reset the detector reference point. The camera automatically activates the flag when the software determines there has been too great of a shift in measured temperatures of pixels. A minimum time of 12 s between flag activations was implemented. A notable difference here is that the image recorded during the flag activation is kept static, as opposed to the CLAMIR camera, where the recorded image goes blank during this time.

By combining the perspectives of all three cameras, a constant view of the melt pool can be ensured, accounting for interruptions from camera autoshutters, or self-obscuring of the melt pool by edges of the manufactured parts. Further, the detection of features in the data becomes more significant if validated by multiple cameras rather than a single camera, which may be subject to noisy signals due to the spatter and variable nature of the melt pool itself.

### Micro computed tomography

The completed samples were cut from the bulk substrate and interrogated by micro-CT in their entirety to reveal internal features. A Zeiss Xradia 515 (Carl Zeiss AG, Oberkochen, Germany) with a tungsten on diamond window X-ray source (140 kV, 10 W), a HE18 source filter, and a flat panel detector was used to image the samples, achieving a pixel size of approximately 18–19 μm, depending on the specific sample.

### Topographical imaging

Topographical images were acquired from some of the samples using a Keyence VHX-5000 (KEYENCE CORPORATION OF AMERICA, Itasca, U.S.A.) 3D Digital Microscope. Topographical images are acquired via a vertical stitching method, where images are acquired over a known vertical range and combined to form a height map.

## Data processing

A custom data processing algorithm was designed and implemented in MATLAB software to track changes to the position, shape, and size of the melt pool from the output of three sensors.

### Target features

The features extracted from the video recordings include:

- The position of the meltpool centroid with respect to the image x- and y-coordinates.
- The equivalent area of the melt pool present in the frame of the image (corrected for perspective distortion).
- The eccentricity of an ellipse fitted to the melt pool object.
- Orientation of the fitted ellipse major axis respective to the image x-axis
- The dimension of the melt pool along the x-axis of the frame for both off-axis cameras, and the dimension in both x- and y-axes of the coaxial CLAMIR camera.

**Figure 4:**
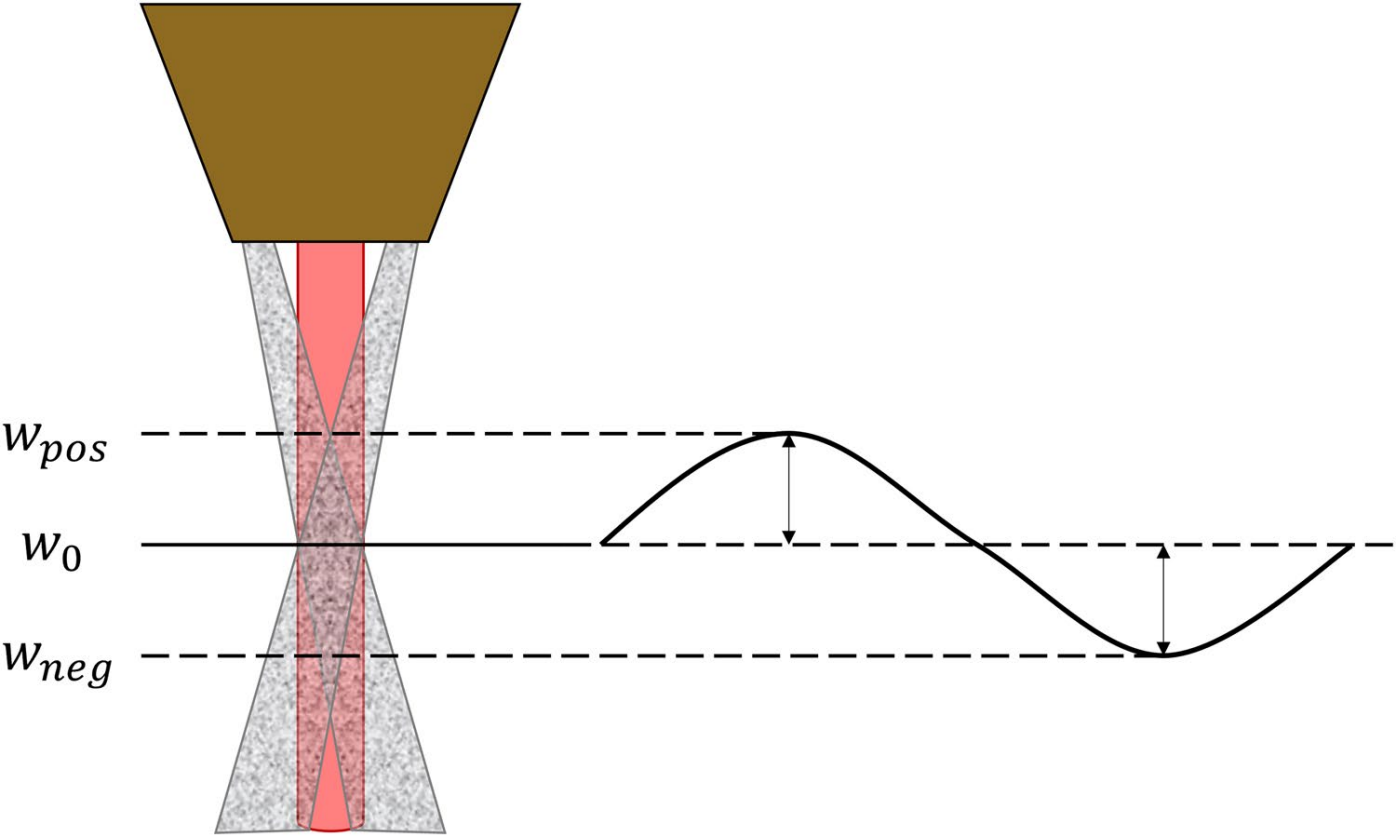
Deviations in the workpiece surface result in positive and negative displacements from the ideal working distance.

The melt pool centroid position was used to extrapolate the vertical displacement of the current deposition plane in relation to the pre-defined working distance. As shown in Fig. 4, an initial working distance, ${w}_{0}$, is programmed into the deposition path. During multi-layered depositions, the nozzle is programmed to raise by a set increment for each successive layer. When the height increment differs from the deposited bead height, the part surface will deviate from the initial working plane, presenting a new working distance, ${w}_{pos}$, or ${w}_{neg}$. In both scenarios, the capture efficiency is reduced. For the case of ${w}_{neg}$, this can result in a further reduced deposition height and a run-away effect with an increasingly negative displacement from the initial working plane^35^. However, in the case of ${w}_{pos}$, the reduced deposit height (as a result of reduced capture efficiency and, or, over-melting) can prevent the part from growing too tall and impacting the deposition nozzle. This results in a stable over-build condition, and is often a desirable build state within which to operate^35^.

When monitoring with an off-axis camera, the displacement of the working distance from the initial working distance can be calculated using Eq. (1), if the assumption is made that changes in the melt pool centroid position correspond to shifts in the workpiece surface. It is hypothesised that the centroid of the melt pool will increment vertically every consecutive layer by the thickness of the deposited layer and, therefore, will provide a method for tracking the change in layer height with respect to the focal plane, defined as the average vertical position of the first layer.

In the case of the melt pool dimensions, only the x-axis is considered in the Optris cameras due to both perspective distortion and potential self-interference along the y-axis, but both can be extracted. Further, the simultaneous measurement by both Optris cameras means that the un-distorted length of the melt pool can be measured by one camera, whilst the second measures the un-distorted width. However, in this study, only the positional features were investigated for the purpose of detecting geometric deviations from the part design.

### Video processing algorithm

**Figure 5:**
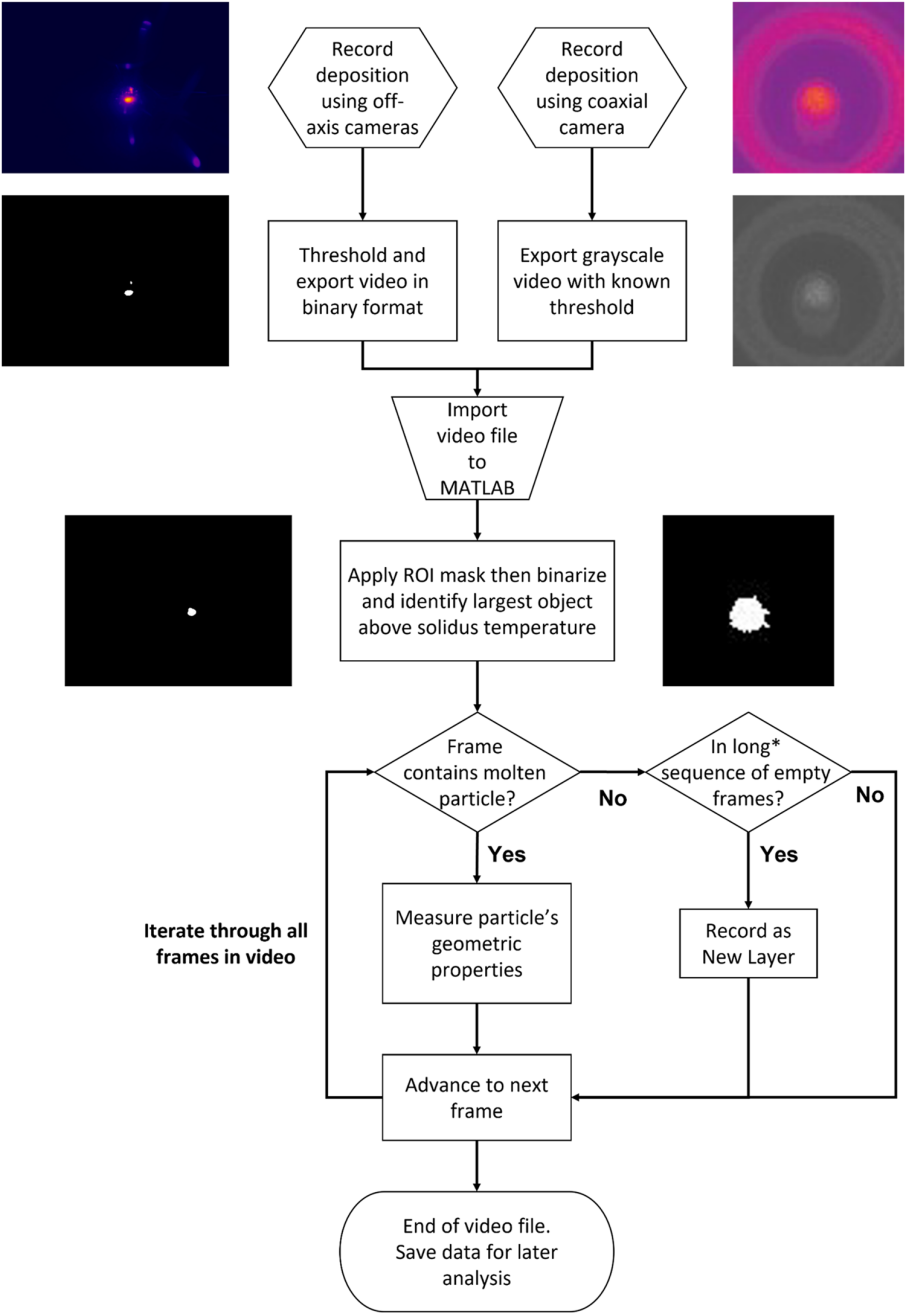
Flowchart describing the algorithm used for processing the video data to vectorised melt pool metrics. *If a frame is in a long sequence of empty frames (typically > 0.5 s equivalent) this indicates the laser was in an ‘off’ state for inter-layer cooling. This step was necessary due to coaxial camera feed being interrupted by the ‘autoshutter’ setting, and this interval time was not always clearly defined.

The video processing algorithm used to extract the above features is summarised by the flowchart in Fig. 5. Additional geometric features could also be extracted through modification of the algorithm in future studies. The signal processing was conducted after the printing was completed. However, there is a potential for this procedure to be automated and performed in real-time to permit a possible closed loop control in the future.

To determine the thresholding value for each of the camera files, a grayscale intensity profile was extracted from the longitudinal axis of the melt pool. The tail of the melt pool exhibits a characteristic cooling curve, interrupted by a flatter ‘shoulder’ with a decreased thermal gradient compared to the rest of the curve. The lower vertex of this shoulder is taken as the solidus temperature, 1600 °C for Ti6Al4V^32^, and used to binarise the frames. A more detailed explanation of this process can be found in the Supplementary Note: Image Processing Algorithm.

Once binarised, frames were cropped, and a Region of Interest (ROI) mask applied to eliminate any extraneous input. The largest object, termed ‘particle’ by the software interface, is selected using the MALAB ‘bwpropfilt’ function to select the largest object by area. This isolates the melt pool from additional spatter. It is assumed here that the largest particle present in a frame will be the persistent melt pool, which is true in most cases. However, the sporadic nature of spatter particles means that a particle may fly towards one of the off-axis cameras, momentarily becoming larger in the field of view than the melt pool. This creates noise in an individual camera recording but is overcome through the availability of multiple sensors, enhancing the obtained result.

Once the content of the frames has been reduced to the single largest particle, the geometric properties of the melt pool are analysed using the ‘regionprops’ function. Using this function, the centroid, bounding box, and area are returned. In addition, an ellipse is fitted to the particle, and the eccentricity, orientation (angle between major axis and image horizontal axis), and major and minor axes lengths are returned. These values are then further processed to extract the metrics identified previously. Further information regarding the implementation of these MATLAB functions can be obtained from the relevant documentation^36^.

The algorithm progresses frame by frame through the video sequence, extracting metrics at each step. Long sequences of frames with no particle present can indicate the laser has turned off for the inter-layer pause, while short sequences may indicate the camera has activated the ‘flag’ or ‘autoshutter’. Therefore, sequences of frames that have no melt pool present for longer than 0.5 s are considered to demark start and end points for layers, and the intervening empty frames are discarded. Data is then automatically separated into a layerwise structure array and stored so that the metrics can be analysed later. A brief discussion of the computational efficiency for this algorithm is provided in Supplementary Note: Image Processing Algorithm online.

## Results and discussion

### Manufactured morphology

**Figure 6:**
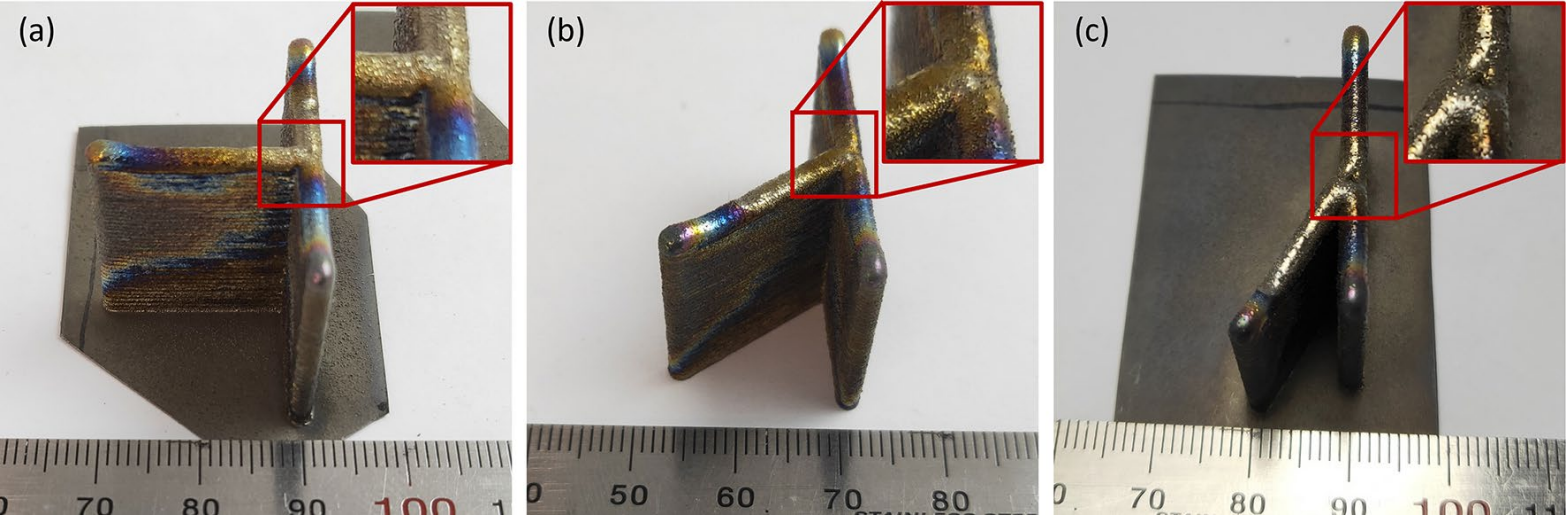
As-built morphologies of samples (**a**) IW1, (**b**) IW4, and (**c**) IW7. Inset in (**c**) shows the location of depression formed during deposition.

Photographs of the manufactured parts are presented in Fig. 6 for the three intersection angles with a design height of 20 mm.

Samples manufactured with a 90° intersection, Fig. 6a, present a largely even surface with raised wall-ends and a flat junction region. Samples manufactured at 60°, Fig. 6b, and 30°, Fig. 6c, similarly present the raised wall ends and even surfaces between the junction and wall ends. However, for all acute-angled samples, a depression forms in the centre of the junction, paired with a bulge on the interior of the angle. The depression is deepest for the 30° angled samples, Fig. 6c, and exhibits an accumulation of adhered particles.

These structural features correlate with the three primary locations of interest, shown in Fig. 1, and can be classified as: the middle regions, the wall turn-around points, and the junction region. The peculiarities of these structural features occur respective to the intersection angle only rather than the design height, with the 5 mm and 20 mm samples exhibiting near-identical surface topography. This indicates that the initiation of each feature class begins early in the build process. The middle regions, where the melt pool is traversing in a straight line at constant speed, exemplify a steady-state deposition with little influence from other portions of the build and are akin to the production of a two-track wide thin wall with 50% overlap. The turn-around points are situated at the end of each wall segment where the deposition head reverses direction and returns for the adjacent track, increasing the time spent depositing material in the vicinity resulting in a build-up of material, and heat, at these locations. The junction of the walls is the third and most variable of the regions for analysis.

Unlike the middle regions and turn-around points, which are similar for all samples, the junction region exhibits different morphology dependent on the angle of intersection between the walls. The DCAM slicing software used to generate the toolpath for the samples from the CAD model automatically creates fillets between walls, and therefore, depending on the angle of intersection, the fillets alter the degree of overlap the laser and powder spots have in the junction region. In the 90° samples, the overlap is evenly dispersed, and there is minimal height variation in the junction. In the case of the 60° sample, the fillet on the interior of the angle results in a bulge of excess material compared to the surrounding steady-state region. Further, a depression also forms in the centre of the junction, where there is now reduced coverage by the laser and powder spot. This bulge and depression pair is accentuated in the 30° sample, where the deeper depression also contains an accumulation of partially melted particles, similar to those found adhering to the sides of the thin walls. This indicates that insufficient energy is being applied to this region to effectively melt these particles, leading to the formation of internal defects within this region.

### Junction void formation in filleted samples

**Figure 7:**
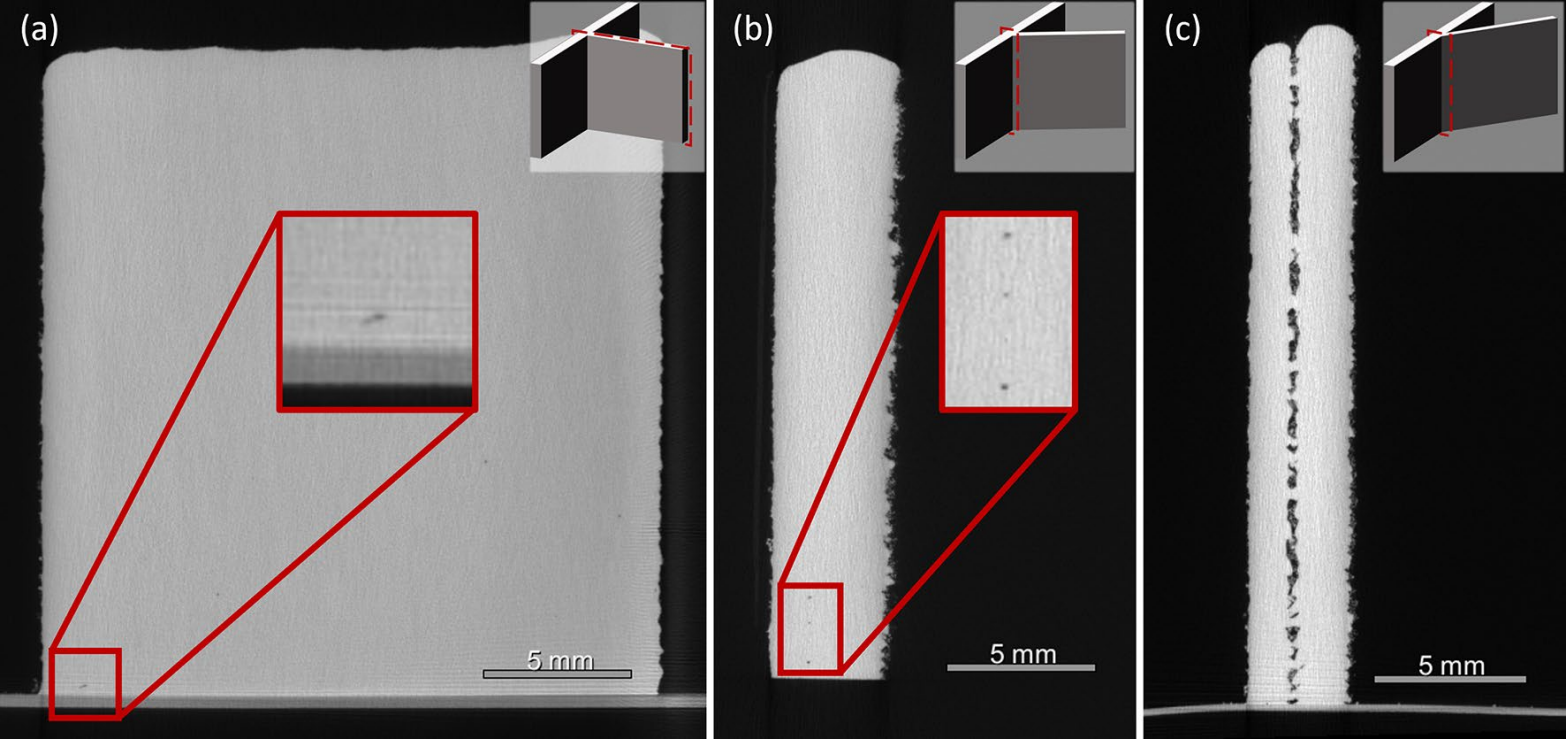
Comparison of porosity located in the junction regions of the (**a**) IW1, (**b**) IW4, and (**c**) IW7 samples. The IW7 sample shows near-continuous porosity throughout the central axis of the junction.

Micro-CT scans were obtained for all manufactured samples. A representative cross-sectional image from the junction region of samples with three different intersection angles is provided in Fig. 7

While some pores are formed in the 90° and 60° samples, Fig. 7a,b, these are typically disperse, rounded, and small in both number and size compared to those formed in the 30° samples, Fig. 7c. While the largest pore detected in the 90° samples was approximately 180 μm in length, the pores in the 30° samples constitute a near continuous void down the central axis of the junction region. Some also present irregular morphologies, suggestive of lack of fusion defects. The presence of adhered particles in the depression of the 30° samples, but not the 60° samples indicate insufficient heat for full melting to occur, therefore resulting in the lack of fusion porosity seen in Fig. 7c. We hypothesise that this occurs due to the automatic filleting of the toolpath reducing the laser overlap in the centre of the junction at narrower intersection angles and demonstrate in “Elimination of junction defects using a modified toolpath” section that it can be avoided.

The porosity in the junction of the 30° sample occurs in the same location as the depression observed in Fig. 6c. This suggests that the surface depression observed in these samples is an indication of internal defects caused by the depletion of powder and laser energy in the centre of the junction. However, it is also clear that the mere presence of a depression does not constitute a formation of voids. The 60° samples exhibit a shallow depression, but there is only a slight increase in junction porosity from the 90° samples. The actual formation of internal defects must be otherwise confirmed, either by post-deposition inspection (e.g., by micro-CT as seen before) or by process monitoring.

### Feature identification by monitoring system

**Figure 8:**
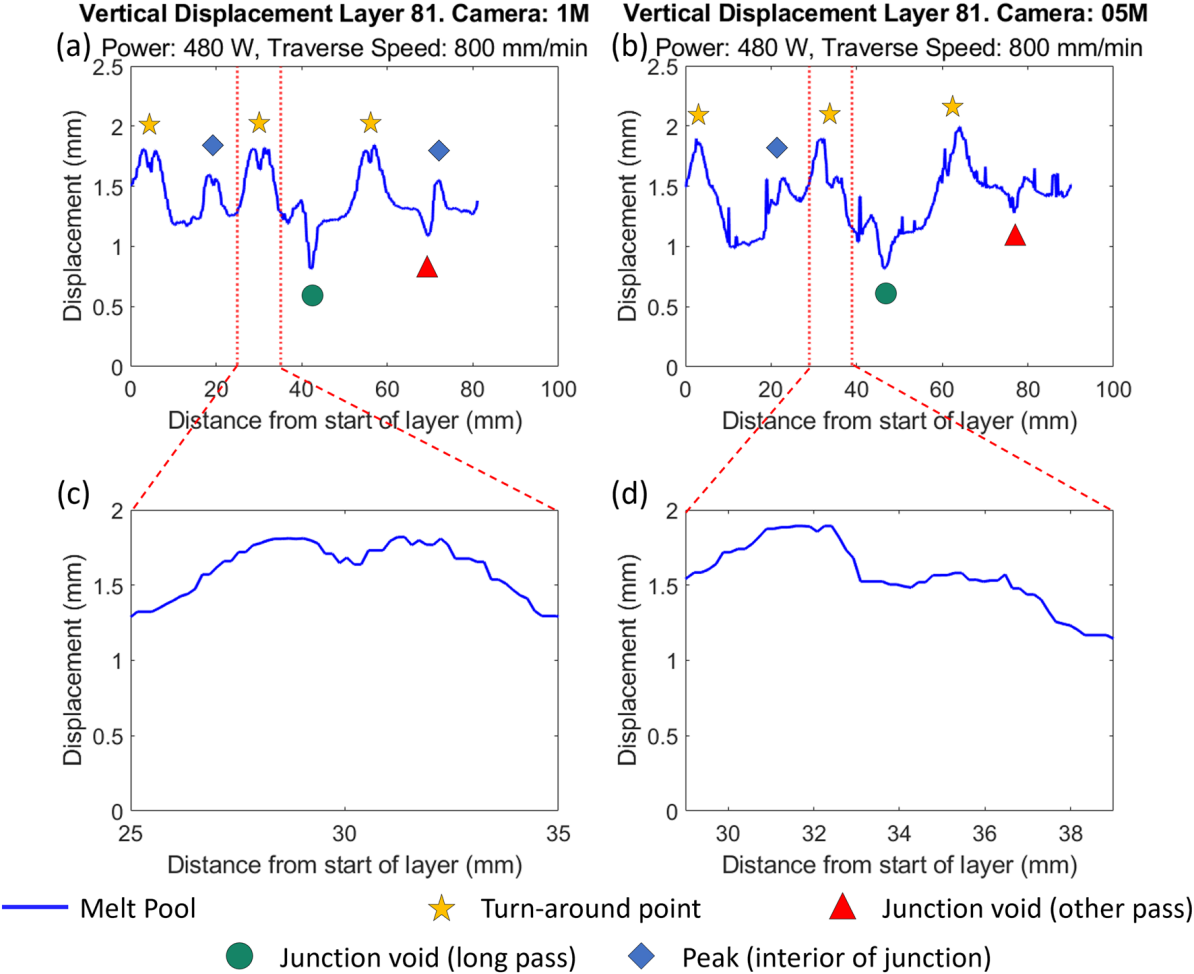
Layerwise data obtained from the final layer by the two off-axis cameras during the manufacture of sample IW7. Key features have been identified and labelled. (**a**) Vertical displacement from focal plane as measured by the 1M camera (viewing along j-axis). (**b**) Vertical displacement from focal plane as measured by the 05M camera (viewing along i-axis). (**c**) Example of symmetric turn-around peak for vertical displacement. Occurs when camera is perpendicular to wall end. (**d**) Example of asymmetric turn-around peak. Occurs when camera is aligned with wall end.

The vertical displacement, as measured by each of the two off-axis cameras, is plotted as a function of the distance from the starting point of each layer in Fig. 8.

Characteristic features can be identified in the displacement graphs shown and ascribed to the three locations of interest of the intersecting wall samples. Firstly, the mid-regions of the thin walls show a predominantly flat baseline value, as might be expected from a steady-state deposition and can be seen in Fig. 8a,b. Similar regions of constant baseline value are also observed in the melt pool area measurements by the coaxial camera (Supplementary Fig. S1), although the noise from this sensor is higher. It is clear from the off-axis cameras that this steady-state deposition occurs at a positive displacement from the plane of the nozzle working distance. These overbuilds in the mid-regions were measured using the two off-axis cameras for a 5 mm span between the wall ends and the junction region along the longer wall and compared with measurements taken from the micro-CT scans in the same positions. The average overbuild values are provided in Table 3. It can be seen that while the off-axis cameras provide similar measurements, the micro-CT data provides an overbuild measurement that is consistently double the estimation of both off-axis cameras.

**Table 3:** Overbuild for a 5 mm span in the mid-regions along the longest wall.

Measurement	Design height and uncertainty (1 standard deviation)					
	5 mm	St. dev.	10 mm	St. dev.	20 mm	St. dev.
05M camera	0.89	0.10	1.05	0.24	1.15	0.17
1M camera	0.80	0.06	1.10	0.11	1.14	0.11
Micro-CT	1.85	0.27	2.19	0.23	2.28	0.26

The primary reason for the discrepancy between the overbuild measurements by micro-CT, and the estimations by melt pool tracking may be the evolution of the build shape. When the baseline value was taken for the centroid in the first layer, the substrate was flat, and the melt region was constrained to its surface. As the build progresses, there is potential for melting of prior layers, shifting the centroid down relative to the actual build surface. Therefore, while the centroid tracking does inform the surface topography well, it may not fully capture the actual over-build estimate without adjusting for this offset. For the samples produced in this study, a constant value correction factor of 2.1 accurately accounts for the offset. However, further work may be required to elucidate further relationships between the centroid position and the surface height increment.

Overall, the trend is to an increasing overbuild for taller design heights, but it is expected that this trend would not continue to increase without limit, instead reaching a stable overbuild condition as discussed by Donadello et al.^26^.

There are two other classes of features evident in Fig. 8a,b, as peaks and troughs interrupting the steady-state. The turn-around peaks are consistently the highest values of displacement, with roughly equivalent values amongst all three turn-around points, in the measurements from the off-axis cameras. They are also characterised by the largest area values obtained from the coaxial camera (Supplementary Fig. S1). On closer inspection, these peaks can be further classified by their characteristic shapes, being split into doublets which can be symmetric or asymmetric in shape, as illustrated in Fig. 8c,d respectively. Asymmetric peak splitting results from self-blocking, which occurs when the melt pool is travelling along the camera’s optical axis. As the melt pool travels away from the camera, the hottest and largest part of the melt pool is partially obscured by the tail of the deposited track, resulting in a lower sub-peak. The local minimum is then formed as the melt pool reaches the extreme of the wall and partially drops below the edge, becoming further obscured from the camera and experiencing a decrease in vertical displacement. As the melt pool reverses direction and moves towards the camera, the melt pool is now at its largest in the camera’s view, and more material has been deposited, resulting in the higher sub-peak in the doublet. Similarly, the symmetric peak splitting also occurs because the melt pool partially dips over the edge of the wall at the extremities, but in this case, the camera’s optical axis is aligned perpendicular to the melt pool’s traverse direction. Hence, there is little difference in the view of the melt pool position, and both peaks present equivalent values.

Associated with the junction void formation in filleted samples described in “Junction void formation in filleted samples” section, smaller single peaks can also be present in some samples. These typically indicate the presence of a bulge on the interior of the angled intersection between walls and hence are not present in the 90° samples and most strongly evident in the 30° samples. Depending on the viewing camera and the traverse direction, this peak can be isolated or adjacent to a sharp trough. This trough occurs in samples that exhibit a depression in the centre of the junction region as the melt pool moves down into this depression. The order of peak and trough in these coupled signals indicates the traverse pathway of the laser spot at that point, whether it is passing the bulge or the depression first.

The appearance of the trough is also seen to be more pronounced in the view of the off-axis vertical displacement graphs, Fig. 8a,b, than the coaxial area graph (Supplementary Fig. S1). This indicates that, while the area of the melt pool may decrease when passing the depression, this change is small compared to the sharp apparent increase in area at turn-around peaks, which is due to increased residence time of the laser and higher elevation at these points. When passing the depression, there is no change in residence time compared to the mid-regions, only the reduction in vertical displacement, which has a small effect on the apparent area for the coaxial camera. This illustrates that, while the coaxial camera is able to monitor the geometry of the melt pool without perspective distortion, and also able to clearly show the location of turn-around points, other geometric features are less evident than in recordings by the off-axis cameras.

### Early detection of defects by height monitoring

**Figure 9:**
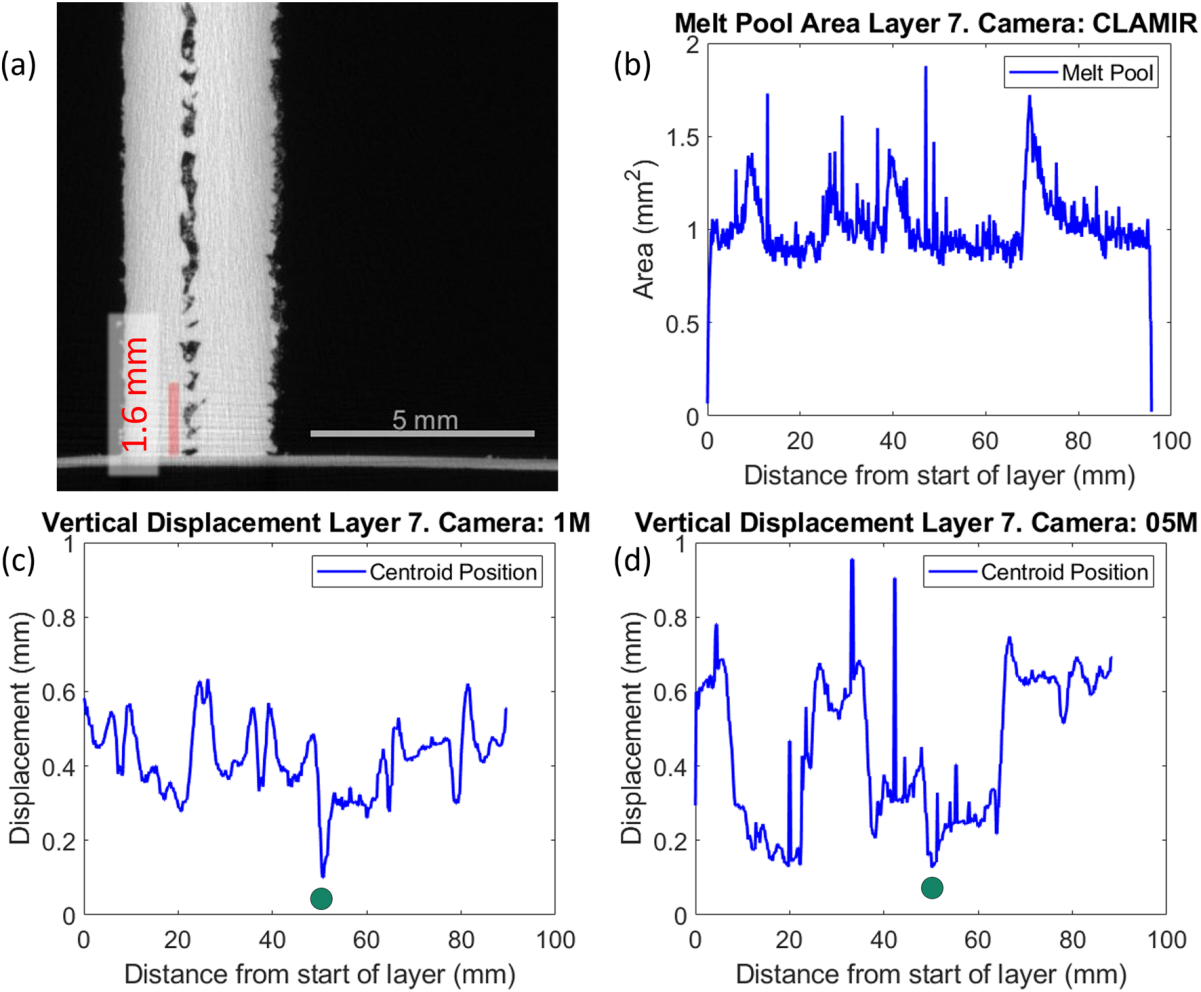
Early detection of defects in sample IW7 (Power: 480 W, Traverse Speed: 800 mm/s). Vertical cross-section of junction with micro-CT scan (with approximate height of sample at layer 7 indicated) (**a**), melt pool area measurement by CLAMIR camera (**b**), vertical displacement from focal plane in 1M camera (**c**), and 05M camera (**d**) all at layer 7. The clearest indication points for the depression are marked with a green circle in the vertical displacement graphs.

These features in the camera signals, and their connection to physical phenomena in the manufactured samples suggest the potential for early detection of void defects. By examining the signals generated by the camera feeds, it is possible to identify the occurrence of similar data features at layers lower down in the sample and, therefore, earlier in the build. Of particular interest is the presence of the depression trough, which is indicated in the previous section to correlate with the formation of voids within the junction region. As is shown in Table 4*,* the depression trough can be identified at an early layer number with respect to the total number of layers in the sample. It should be noted that the ‘Layer distance’ reported here is the distance the melt pool has travelled since the camera was first able to detect it, and that the start position of each layer is different to that of other layers. An example of early detection is shown for the sample IW7 in Fig. 9.

**Table 4:** First instances of trough feature identification in off-axis camera data for three 30° samples.

Sample name	Layer number	Camera name (M)	Layer distance (mm)	Vertical displacement (mm)	Mean displacement for layer (mm)
IW7	7/81	1	50.65	0.10	0.42
		05	50.14	0.13	0.44
IW8 (two points of detection)	5/41	1	23.3	0.22	0.28
			48.79	0.02	
		05	23.80	0.02	0.30
			50.14	0.02	
IW9	7/21	1	44.57	0.07	0.38
		05	47.10	0.06	0.41

Table 4 shows that it is possible to detect the presence of the depression at an early point during the manufacture of these samples and that there appears to be a preferential scan direction for identifying that trough with both the off-axis cameras. The earliest detection always occurs on an odd-numbered layer, with the laser travelling counter-clockwise; therefore, the 05M camera is viewing the tail of the melt pool as it traverses the uninterrupted scan of the long wall section. Thus, as the melt pool reaches the depression, the front of the melt pool is partially obscured, and the drop in centroid position is clearer to the camera. On even-numbered layers, the melt pool would be traversing towards the 05M camera, and the bright front of the melt pool increases the size of the detected particle, reducing the sensitivity of centroid position tracking.

The micro-CT scan of the sample, Fig. 9a, confirms that a void has formed at the location of the depression that was detected. In the case of sample IW7, the first detection of the depression occurs at layer 7, where the expected height of the sample, and therefore the focal plane, has increased to 1.5 mm above the substrate. The trough detected here by both the 1M and 05M cameras, Fig. 9c,d shows a vertical displacement of 0.1 mm above this plane. As illustrated in Fig. 9a, a pore is located at 1.6 mm above the substrate, however, this pore is not the largest, nor the first to occur up until this point. This suggests there may be a minimum height required for the body of the sample to reach before a depression is detectable. Figure 9b shows the data collected by the coaxial camera during the same layer, and while the pore defect is not clear in this data, the sharp peaks seen correspond with the turn-around locations where the melt pool is larger in the view of the camera.

### Camera limitations

In understanding the information obtained from the monitoring system, the cameras’ limitations and viewing conditions must be acknowledged.

One important limitation of the coaxial camera is that the nozzle aperture restricts the field of view (FOV) to a region approximately 5 mm in diameter. The FOV can therefore dictate the laser spot diameter used for processing. Based on internal studies, the laser spot size used was 1.5 mm in diameter, allowing the melt pool tail to be visible within the aperture under most conditions. An additional limitation of the coaxial camera is that IR light can be reflected and scattered from the inside of the nozzle, creating a ring of light around the desired region of interest, as shown in Fig. 2b, which can interfere with image analysis. This also imposes a soft limit on the energy density applied to the process zone. Higher energy densities result in higher melt pool temperatures and larger sizes. This also increases the amount of IR radiation reflected by the inside of the nozzle and further decreases the FOV for the camera. Similarly, longer melt pool tails can also be truncated. Therefore, while the undistorted coaxial view is advantageous for accurate melt pool area and geometry measurements at smaller melt pool sizes, any extension of processing parameters outside a limited range requires the addition of at least one other off-axis camera.

The off-axis cameras are subject to perspective distortions, as discussed in “Geometric corrections” section, which must be accounted for. Further, observing the melt pool from an angle can result in temporary or partial blocking of the melt pool during depositions. Blocking can result from other objects within the build environment, such as the deposition nozzle, or other areas of the same sample.

The problem with perspective distortion in one off-axis camera can be illustrated by comparing the melt pool length measured in profile (melt pool traverse direction aligned with camera axis) and in landscape (melt pool traverse direction perpendicular to camera axis). Take the first layer, to eliminate effects of part over-growth on measurements, of the IW2 sample as an example. When the melt pool is in profile, moving towards the Optris 1M camera, the mean length over a 10 mm stretch is approximately 1.9 mm (correcting for perspective angle), while the undistorted view of the Optris 05M and CLAMIR measure this value to be 1.6 mm and 1.3 mm respectively. Similarly, in the same orientation, the melt pool width measured in undistorted profile by the Optris 1M camera is 1.3 mm, agreeing with the CLAMIR measurement of 1.3 mm, while the distorted view by the Optris 05M camera measures the width at 2.4 mm.

In general, if self-blocking is not occurring (i.e. deposition behind an adjacent track previously deposited) the measurement of length or width along the image y-axis for the angled cameras often provides an overestimate of the same values measured by the companion camera along a non-distortion affected camera. However, this becomes increasingly complex with the geometry of the component, hence reinforcing the necessity of having multiple cameras to capture measurements along multiple axes.

### Elimination of junction defects using a modified toolpath

**Figure 10:**
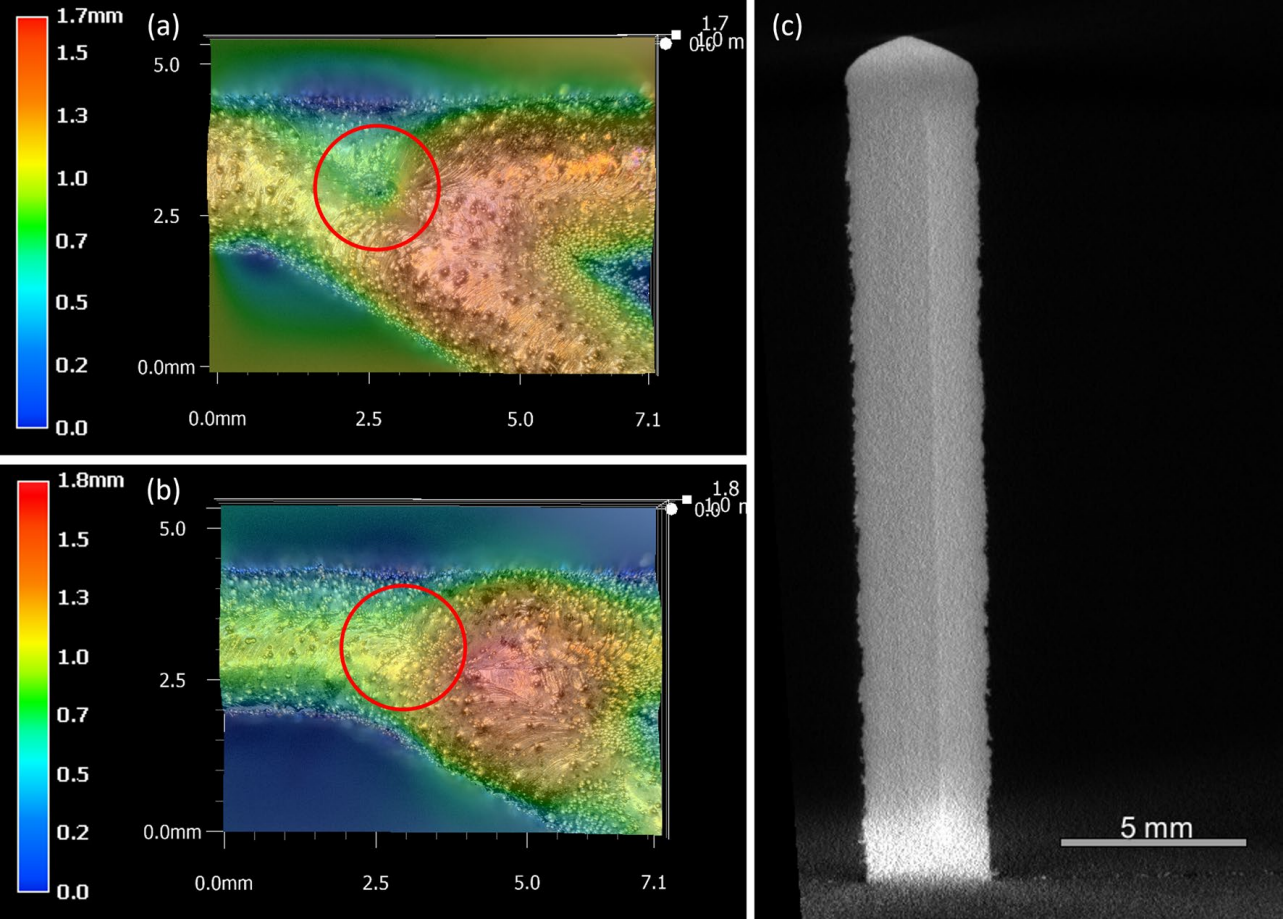
Comparison of junction region in 30° samples with topographical images of (**a**) original, IW7, and (**b**) modified, IW7-Mod, toolpaths as imaged by Keyence VHX-5000 3D digital microscope at × 50 magnification. Micro-CT image showing the cross-section of the junction region in IW7-Mod without the same porosity as in IW7. The junction region is highlighted in both (**a,b**) showing the absence of the depression in the IW7-Mod sample.

To confirm that camera detection was not an artifact of intersection angle alone, and that the formation of the junction void defects was occurring due to the toolpath planning of the narrow-angled intersections, a further set of samples were produced with modified toolpaths that eliminated the fillets at the intersection. This modification should theoretically maintain a more consistent level of laser spot overlapping at the junction compared to the original samples. These modified samples were all produced to the same design height of 20 mm, as only the formation of junction defects was being investigated, and the previous samples had shown that these were unaffected by sample design height. Topographical images were obtained for the junction regions using the Keyence digital microscope, and micro-CT was performed on the samples with the results presented in Fig. 10.

The modified toolpath samples presented a surface similar to the original samples, except for the absence of the depression at the centre of the junction, as can be seen in a comparison of Fig. 10a,b, although a bulge was still seen to form on the interior of the angled intersections for the 60° and 30° samples.

An inspection of the signals extracted from the monitoring setup also shows no indication of depression formation in the modified toolpath samples but does indicate the formation of a bulge that still occurs in the 60° and 30° samples. When the micro-CT results were inspected the absence of defects in the junction region of the 30° samples is ultimately confirmed, Fig. 10c. Together, the observations of these modified samples illustrate two important findings.

First, the formation mechanism for the junction void defects is found to be the result of the toolpath depleting the junction of both laser irradiance and feedstock, resulting in a reduced deposition of material. Therefore, toolpath planning must consider the effects of reduced overlapping at junction regions in components with complicated geometries. Intersecting angles of less than 60° may result in increased porosity levels if corner fillets prevent full overlap of pathways. As fillets are a method of minimising sharp corners in geometries, it is often not practical to remove these from structural components, otherwise parts may experience an increased risk of failure due to fatigue or cracking^37^. Hence, other methods of ensuring full melting at narrow-angled intersections must be considered, depending on the intended design. These may include incorporating an additional interior scan at the centre of the junction, so long as this does not unduly increase the heat and material accumulation at this location. Alternatively, it might be suggested that the toolpath should be altered to increase the distance from the intersection point at which the fillets are implemented, reducing impacts of increased laser residence time in the intersection region. This could be coupled with an internal scan to further reinforce the junction; however, this would come at the cost of increased time, weight and material.

Secondly, the detection of this depression in the image-based tracking system is shown to be irrespective of intersection angle, and no artifact in the imaging could be considered as providing a false correlation between angle and depression signalling.

## Conclusions

This work presents a multiple axis infrared vision-based system for in-situ monitoring of Laser Directed Energy Deposition using three cameras. A video processing framework for tracking the evolution of the melt pool throughout the deposition to extract displacement and geometric information is described. It is shown that fluctuations in vertical displacement monitoring can detect the formation of structural features, such as depressions and bulges, in thin-walled structures containing intersecting walls of various angles. The advantages of multi-sensor monitoring in detecting and identifying these features is demonstrated, with features recognised independently across multiple sensor configurations, whilst also providing differing process information.

It is demonstrated that reduced laser spot overlap, due to automatic filleting in toolpath planning, creates regions of reduced deposition quality exhibited by surface depression, resulting in internal defect formation, most prominent in samples with a 30° intersection. The prevalence of this internal porosity is proven using micro-CT. Likewise, the modification of the toolpath to remove automatic fillets is shown to eliminate this internal porosity. The signals corresponding to the surface depression are identified by the algorithm and highlight the system’s capability for early defect detection, for instance, in samples with a 30° intersection. This enables the integration of an in-line early warning system to detect defect and geometric feature formation, which will be implemented in the future work. Finally, it is recommended that toolpath planning for complex geometries with narrow intersection angles consider the effects of filleting on laser irradiance of parts.

Future works will extend on the results of this study by analysing the relationships between other extracted metrics and potential defect states in deposited samples. With streamlining and down-sampling of data transfer, this system may be realised for real-time monitoring.

### Supplementary Information

Supplementary Information.

## Data Availability

The datasets generated and/or analysed during the current study are not publicly available as they are currently being evaluated as part of further studies and also due to the proprietary file format of many of the recorded video files. Data will be made available from the corresponding author on reasonable request.
