# Supplementary material for: Defect detection by multi-axis infrared process monitoring of laser beam directed energy deposition
Source: Sci Rep. 2024 Feb 16;14:3861. doi: 10.1038/s41598-024-53931-2 (PMC10869730; doi:10.1038/s41598-024-53931-2)
Supplement: Supplementary file 1 — Supplementary Information. [file 41598_2024_53931_MOESM1_ESM.docx]

# Supplementary Note: Image Processing Algorithm

The video processing is predicated on particle analysis in binary frames, and the thermal threshold value needs to be determined for each camera. According to the blackbody model of thermal radiation, an object will emit radiation with a wavelength distribution governed by temperature. By monitoring the intensity of specific wavelength bands, it is possible to determine the temperature of an object using the Stefan-Boltzmann law, equation (1). Most real objects do not behave as blackbodies and have complex relationships between the emissivity of the surface and its temperature and physical or chemical state that are often poorly understood. A common simplification is to use the graybody assumption and determine an emissivity value for the surface. This value can depend on many factors, including absolute temperature^1^, viewing angle^2^, surface condition, oxidation state and more^3^. In this study, the graybody assumption was used, and the effective emissivity of the system was determined for both Optris cameras by use of the solidification temperature of Ti6Al4V, similar to the calibration process used in^4^.

Using the camera, one measures the temperature of the solidus at an emissivity of 1, then imposes the known temperature of the solidus, and the emissivity which produces the desired temperature response of the camera can be calculated.

$$\begin{aligned} P=\epsilon\sigma AT^{4}\#\left( 1 \right) \end{aligned}$$

$$\therefore\epsilon_{1}\sigma AT_{1}^{4}=\epsilon_{2}\sigma AT_{2}^{4}$$

$$\begin{aligned} \therefore\epsilon_{2}=\frac{\epsilon_{1}T_{1}^{4}}{T_{2}^{4}}\#\left( 2 \right) \end{aligned}$$

Where $P$ is the radiated power, identical in both cases as the same recording is used, $\epsilon$ is the emissivity, $\sigma$ is the Stefan-Boltzmann constant, and $T$ is the temperature in kelvin.

Taking a still frame from each of the camera feeds that shows a clear image of the melt pool an intensity, or temperature, profile line can be taken longitudinally down the centre of the melt pool. The intensity profile towards the tail portion of the melt pool exemplifies a characteristic cooling curve but exhibits a shoulder that occurs when the liquid solidifies. As crystallisation from the molten state is an exothermic process, the temperature in the melt pool tail plateaus, resulting in this 'shoulder' with a decreased thermal gradient with respect to the rest of the curve. The start and end points of this shoulder represent the liquidus and solidus points. By assuming these occur at the same temperature as Ti6Al4V equilibrium temperatures, 1650°C and 1600°C respectively^5^, the effective emissivity can be calculated using equation (2). In this study, the solidus point has been used as the calibration temperature to determine the emissivity of each system, and as the definition of the melt pool boundary under the assumption that the semi-molten region between 1600-1650°C will still capture feedstock powder. To implement the calibration, the calculated emissivities from the off-axis cameras were applied to recordings such that the temperature measured by the camera at the melt pool boundary now matched the expected value.

In the case of the coaxial camera, where no temperature value is estimated by the software, a greyscale value was obtained from the lower vertex of the shoulder in the cooling curve. This value was found to be consistent across a wide range of process parameters that were tested and was used to threshold the greyscale images.

In the case of the two off-axis cameras, the recordings must be thresholded using the solidus temperature as described above, and the video file exported. The exporting process utilised by this software limits the video file to the refresh rate of the screen being used, 30Hz in this case, and therefore the recording is reduced in playback speed to 0.38 times, to preserve as much of the original captured data as possible. For the coaxial camera, data can be exported directly as a video file with full temporal resolution; in this study, they were exported with 100Hz framerate.

Due to the proprietary recording format of both off-axis cameras, the current system is unable to directly export the IR video footage for video processing. Hence, this study was conducted on pre-recorded depositions. However, to elucidate the potential for future incorporation to real-time, or near real-time processing, the time taken for the extraction of metrics was measured for the IW2 sample. The recording time for this sample was approximately 19.5 minutes (with the largest difference between recordings being 35 seconds). Both Optris cameras recorded at 80 Hz, and the CLAMIR recording was downsampled to 100 Hz for analysis. The individual processing of the recordings took 14:49 minutes for the Optris 1M recording, 13:18 minutes for the Optris 05M camera and 19:52 minutes for the CLAMIR camera. This analysis was conducted on a HP EliteBook x360, with an Intel Core i5-8265U CPU with base frequency of 1.6 GHz, and 8 GB installed RAM. For each recording, the data processing occurs on a comparable timescale to the recording itself, although the total analysis time between cameras is greater than the recording time for the sample. However, the code has not currently been optimised for efficiency and future runtime improvement could be expected. Additionally, when executing real-time analysis, it will likely be necessary to further down-sample the recording framerate, sacrificing fine-grained resolution for improved runtime efficiency.

# Supplementary Figure S1: Melt Pool Area Measurements

The melt pool area of sample IW7 was also extracted, and the data structure compared with the surface topography to identify the correlation of physical structures with the recorded data, Fig. S1.


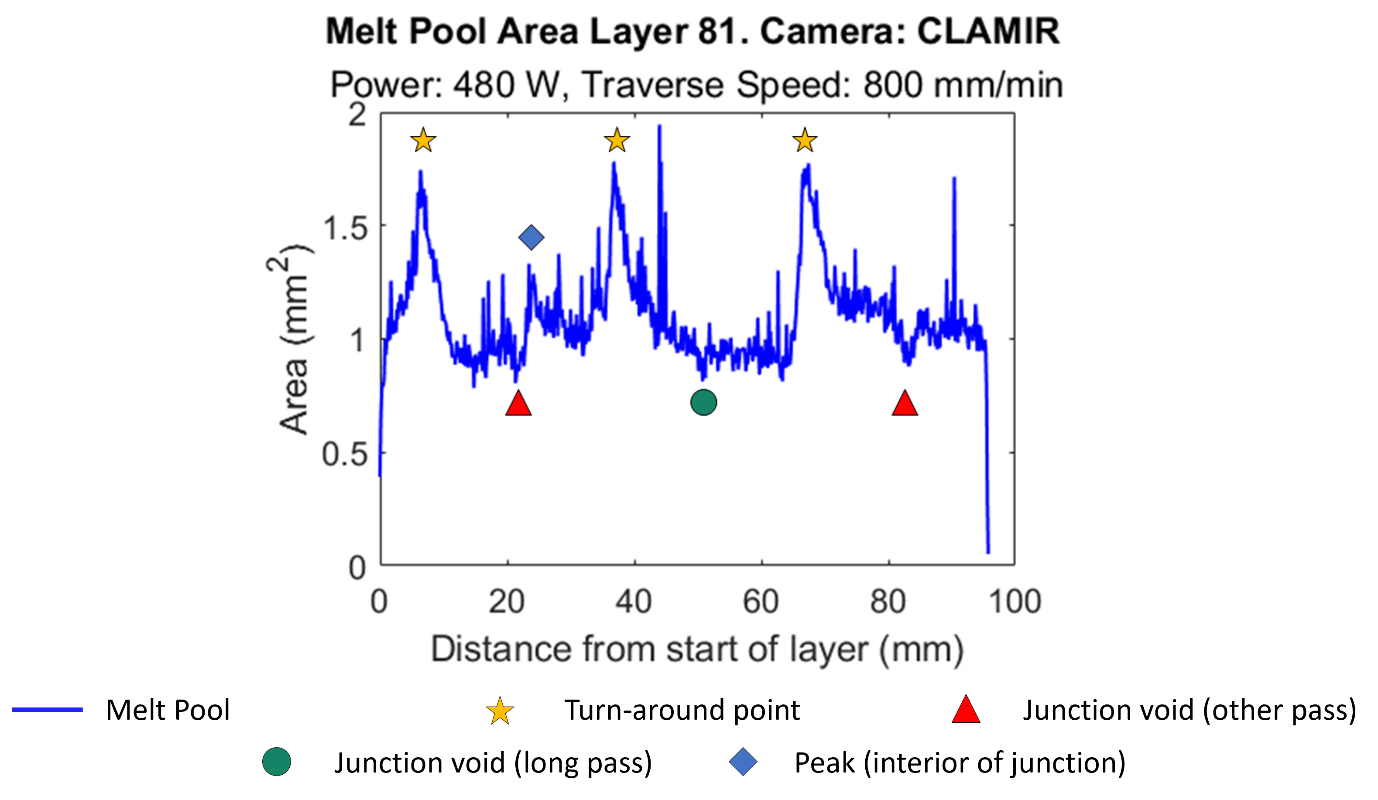


The turn-around points are clearly discernible as a result of the increased residence time, while the peak corresponding to the junction bulge peak is smaller, but still apparent. The troughs that are clearly evident with the off-axis cameras are largely indistinct using the coaxial camera and are more easily identifiable when considering other data sources in combination.

# References

1 Farshidianfar, M. H., Khajepour, A. & Gerlich, A. Real-time control of microstructure in laser additive manufacturing. *Int. J. Adv. Manuf. Technol.* **82**, 1173-1186, doi:10.1007/s00170-015-7423-5 (2016).

2 Peeters, J., Ribbens, B., Dirckx, J. J. J. & Steenackers, G. Determining directional emissivity: Numerical estimation and experimental validation by using infrared thermography. *Infrared Physics & Technology* **77**, 344-350, doi:<https://doi.org/10.1016/j.infrared.2016.06.016> (2016).

3 Felice, R. Pyrometry for liquid metals. *Advanced materials & processes* **166**, 31-33 (2008).

4 Gould, B. *et al.* In Situ Analysis of Laser Powder Bed Fusion Using Simultaneous High-Speed Infrared and X-ray Imaging. *JOM*, doi:10.1007/s11837-020-04291-5 (2020).

5 Boivineau, M. *et al.* Thermophysical Properties of Solid and Liquid Ti-6Al-4V (TA6V) Alloy. *International Journal of Thermophysics* **27**, 507-529, doi:10.1007/PL00021868 (2006).
